# Oxidative Stress Associated with Neuronal Apoptosis in Experimental Models of Epilepsy

**DOI:** 10.1155/2014/293689

**Published:** 2014-12-29

**Authors:** Marisela Méndez-Armenta, Concepción Nava-Ruíz, Daniel Juárez-Rebollar, Erika Rodríguez-Martínez, Petra Yescas Gómez

**Affiliations:** ^1^Laboratorio de Neuropatología Experimental, Instituto Nacional de Neurología y Neurocirugía, Manuel Velasco Suárez, Insurgentes Sur 3877, La Fama, CP 14269, Tlalpan, DF, Mexico; ^2^Departamento de Fisiología, Facultad de Medicina, Universidad Nacional Autónoma de México (UNAM), Avenida Universidad 3000, CP 04510, Coyoacán, DF, Mexico; ^3^Departamento de Genética, Instituto Nacional de Neurología y Neurocirugía, Manuel Velasco Suárez, Insurgentes Sur 3877, CP 14269, La Fama, Tlalpan, DF, Mexico

## Abstract

Epilepsy is considered one of the most common neurological disorders worldwide. Oxidative stress produced by free radicals may play a role in the initiation and progression of epilepsy; the changes in the mitochondrial and the oxidative stress state can lead mechanism associated with neuronal death pathway. Bioenergetics state failure and impaired mitochondrial function include excessive free radical production with impaired synthesis of antioxidants. This review summarizes evidence that suggest what is the role of oxidative stress on induction of apoptosis in experimental models of epilepsy.

## 1. Introduction 

Epilepsy is a chronic neurological disease characterized by recurrent and spontaneous seizures with diverse etiology that affects up to 1% of the world population. The median prevalence of lifetime epilepsy for developed countries is 5.8 per 1,000 and 10.3 per 1,000 for developing countries [1]. Epilepsy is the most frequent neurodegenerative disease after stroke, and according to epidemiological studies, approximately 70–80% of epilepsy patients achieve remission and approximately 30% of this patients present resistance to pharmacological treatment [2]. Status epilepticus, or the condition of prolonged epileptic seizures, is a major neurological and medical emergency that is associated with significant morbidity and mortality [3]. Epilepsy comprises a large number of syndromes, which vary greatly with respect to their clinical features, treatment, and prognosis; several classifications of the seizures (symptoms) and the epilepsy syndromes have been refined with time. Several causes are associated with epileptic seizures, between others, central nervous system (CNS) tumors, neurodevelopmental abnormalities, CNS trauma, and/or inflammation; likewise, a large group of epilepsies have unknown etiology [4]. Temporal lobe epilepsy (TLE) is the most prominent example of acquired and frequent epilepsy; the seizure origin typically involves the hippocampal formation, a structure located in the mesial temporal lobe. Two main types of TLE are generally recognized, mesial temporal lobe epilepsy, which arises in the hippocampus, parahippocampal gyrus, and amygdala, and lateral temporal lobe epilepsy, which arises in the neocortex [5–7]. In TLE associated with mesial sclerosis (MTLE), the hippocampus represents the epileptic focus, while the temporal neocortex is involved in propagation of epileptic seizures in other brain areas [8].

The brain is particularly vulnerable to oxidative damage because of its high oxygen utilization, its high content of oxidisable polyunsaturated fatty acids, and the presence of redox-active metals (Cu, Fe) [9, 10]. Neuronal cells in the brain are highly sensitive to oxidative stress; therefore, the prolonged excitation of neurons during seizures can lead to injury resulting from biochemical alterations and specifically to the role played by the oxidation state. Oxidative stress is defined as an imbalance between the production of reactive oxygen species (ROS), reactive nitrogen oxygen (RNS), and the ability to readily detoxify the reactive intermediates in a biological system [7, 11, 12]. Excessive ROS generation can cause damage of neuronal cells inducing cell death via either an apoptotic or a necrotic pathway [13]. Recent evidence has suggested an intimate link between oxidative stress and mitochondrial dysfunction with the development of neuronal death in diverse neurological disorders including epilepsy. Mitochondrial dysfunction includes bioenergetic failure and increased cytosolic calcium, oxidative stress (excessive free radical production and impaired synthesis of antioxidants, especially glutathione), mitochondrial permeability transition pore opening, and the release of key proteins into the cytosol triggering cell death pathways such as apoptosis [14].

Experimental epilepsy models have been developed to assess the pathophysiology of epileptic seizures and have played a fundamental role in our understanding of the basic molecular mechanism. Experimental animal models can be divided into three categories mainly: (1) experimental seizures induced by chemical convulsants or by electrical stimulation, (2) reflex epilepsies, and (3) idiopathic epilepsies [15]. The most well known and most frequently used are multiple spontaneous recurrent seizures TLE (kainic acid) a glutamatergic agonist, cholinergic agonist pilocarpine (PILO), or model for induction of epilepsy Pentylenetetrazol (PTZ) a tetrazole that is an antagonist of gamma-aminobutyric acid receptors [15, 16]. Many experimental reports have demonstrated the involvement of oxidative stress in seizures associated with brain damage and the mechanisms associated with epilepsy. The aim of this review is to present recent evidence on its role of mitochondrial dysfunction and oxidative stress in the apoptosis induction in experimental epilepsy models.

## 2. Reactive Oxidative Species and Reactive Nitrogen Species

### 2.1. Free Radicals

Oxygen free radicals or, more generally, reactive oxygen species (ROS) as well as reactive nitrogen species (RNS) are products of normal cellular metabolism [9, 17]. Increased oxidative/nitrosative stress generally describes a condition in which cellular antioxidant defenses are unable to inactivate the ROS and RNS; the amount of free radicals is determined in the mitochondrial oxidative phosphorylation chains [18]. It is now well established that mitochondria is the main site of the generation of oxygen radicals; there are many different varieties of partially reduced ROS including superoxide (O^•^^−2^), hydrogen peroxide (H_2_O_2_), and the hydroxyl radical (OH^•^) [19]. The modern use of the term ROS includes both oxygen radicals and nonradicals that easily converted into free radicals (O_3_, H_2_O_2_, and ^1^O_2_). RNS refer to nitric oxide (NO) and molecules derived from NO, such as peroxynitrite (ONOO-), nitrosyl (ON^−^), and nitrogen dioxide (NO_2_) [17, 18]. Ubiquinone, a component of the mitochondrial respiratory chain connecting Complex I with III and Complex II with III, is regarded as a major participant in the formation of O^•^^−2^ by Complex III [9, 20, 21]. The dismutation of superoxide anions by superoxide dismutase (SODs, which are present in both cytosol, copper/zinc-associated isoform and mitochondria manganese-associated isoform) results in H_2_O_2_ production [7, 22, 23].

### 2.2. Nitric Oxide

Nitric oxide (NO^•^) is an abundant reactive radical that acts as an important oxidative biological signalling molecule in biological activities in several physiological processes including neurotransmission, blood pressure regulation, defence mechanisms, smooth muscle relaxation, and immune regulation [9, 17]. The nitric oxide, or nitrogen monoxide, radical (NO^−^) produced by the stoichiometric conversion of L-arginine to L-citrulline via different isoforms of nitric oxide synthesis (NOS) [24]. Three isoforms account for NO^•^ production and include neuronal NO synthase (nNOS; type I), inducible NO synthase (iNOS; type II) which is produced in very large amounts by activating microglia (macrophages), and endothelial NO synthase (eNOS; type III) [25–27].

Excessive superoxide rapidly reacts with NO and forms peroxynitrite (ONOO^−^) which protonated at relevant pH to form peroxynitrous acid (ONOOH); this reaction is much faster than dismutation of superoxide by SOD and would result in decreased NO bioavailability; both ONOO^−^ and ONOOH are potent oxidizers; ONOOH exhibits hydroxyl radical (OH^−^)-like activity [28]. Peroxynitrite is a potent oxidant that can nitrate tyrosine residues of structural proteins; under physiological conditions, ONOOH can react with other components present in high concentrations, such as H_2_O_2_ or CO_2_, and function as NADPH oxidase [28, 29]. Nitric oxide may take part in nitrosylation of proteins; however, peroxynitrite is a highly reactive nitrogen species, which induces tyrosine nitration, lipid peroxidation, and cytotoxicity, including cellular death [26, 30].

An excessive generation of free radicals (ROS and RNS) and decrease of enzymatic antioxidant activity are considered as the main causes of oxidative stress that can result in cellular injury in the form of lipid peroxidation, DNA damage, protein oxidation and disruption of the cell functions, and/or inducing cell death on the CNS. ROS and RNS are involved in both apoptosis and/or necrosis mechanisms for neuronal death.

### 2.3. Mitochondrial Dysfunction

As described above, the mitochondrial electron transport chain contains several redox centers that may leak electrons to molecular oxygen, serving as the primary source of ROS production, which function as second messengers in signal transduction but are also mediators of oxidative damage and inflammation [7, 20, 31]. A neuron uses much of O_2_ it takes up to make, via mitochondria, ATP needed to maintain low gradients (high intracellular K^+^, low Na^+^, very low, and “free” Ca^+^) adequate energy supply by mitochondria is essential for neuronal excitability and neuronal survival [25, 32, 33].

Dysfunctional mitochondria may contribute to increased ROS production and would be unable to maintain optimal mitochondrial calcium (Ca^2+^) levels which consequently can lead to depolarization of the inner mitochondrial membrane potential [21]. The generation of ROS and the release of proapoptotic molecules to the cytoplasm, mitochondrial swelling, and mitochondrial membrane rupture lead to the activation of different modes of cell death. Those changes that affect neuronal calcium homeostasis may be factors that contribute to increase of susceptibility to epileptic seizures associated with mitochondrial dysfunction [7, 34].

### 2.4. Role of Calcium and Mitochondria

Calcium signaling plays an important role in regulating and maintaining normal neuronal function, including neurotransmitter release, excitability, neurite outgrowth, synaptic plasticity, gene transcription, and cell survival. The mitochondria sequester free intracellular Ca^2+^ through several transport systems maintains cell Ca^2+^ homeostasis and serves as Ca^2+^ buffer which regulates the intracellular Ca^2+^ homeostasis; when Ca^2+^ accumulates in the mitochondria, it released in the matrix along with other solutes and this process also accompanied by oxidative stress and depletion of adenine nucleotides [20]. Neuronal increases in calcium can activate a series of enzymes including protein kinase C, proteases, phosphatases, phospholipases, and xanthine oxidase; the last three (phospholipase A2) produce ROS and RNS by triggering an acid arachidonic cascade [25, 35].

**Figure 1:**
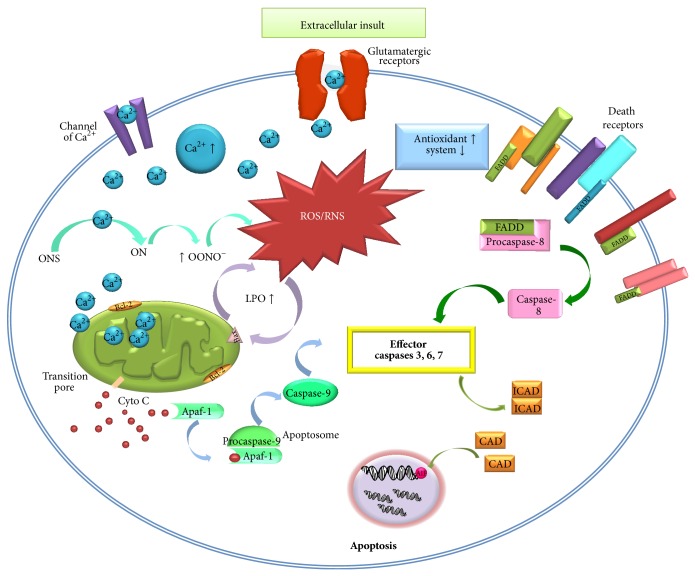
A proposed model of the relationship between apoptosis cell death in epilepsy models. AIF (apoptosis-inducing factor); Apaf 1 (apoptosis protease activating factor-1); Bcl-2 (antiapoptotic protein); Bax (proapoptotic proteins); CAD (caspase activated DNase); ICAD (inhibitor of caspase activated DNase); NOS (nitric oxide synthase), ON (oxide nitric); OONHO^−^ (peroxide nitrite); LPO (lipid peroxidation); ROS (reactive oxygen species); RNS (reactive nitrogen species); Ca^2+^ (calcium); FADD (Fas-associated protein with death domain); Cyto c (cytochrome c).

Mitochondrial Ca^2+^ increase results in enhanced ROS production; between others the potential deleterious effect of ROS production in mitochondria is the facilitation of Ca^2+^-dependent mitochondrial permeability transition pore (MPTP), which can be stimulated to open by excessive concentrations of Ca^2+^ and can also extrude Ca^2+^ [7, 36]. Moreover, Ca^2+^ can active nitric oxide synthase (NOS) and generate NO and peroxynitrite (ONOO^−^), increasing also RNS production [23]. Mitochondrial Ca^2+^ overload triggers the opening MPTP, which can lead to necrosis owing to ATP depletion or to caspase-dependent apoptosis; this confirms the complex interdependence between mitochondria, Ca^2+^, and ROS generation [23]. The release of Ca^2+^ from the endoplasmic reticulum and the activation of the caspase-dependent apoptosis pathway through changes in mitochondrial membrane permeability induce cellular damage (Figure 1) [36, 37].

### 2.5. Cellular Antioxidant Defense

The physiological production of ROS in aerobic organisms requires the presence of a defense system against the effects of these oxidative species. Antioxidants can be divided into two groups: endogenous and exogenous; the mitochondria possess multiple antioxidant defense systems including glutathione, glutathione peroxidase (GPx), superoxide dismutase, catalase, and vitamins E and C [38, 39]. The brain contains reduced levels of glutathione, almost no catalase, and has low concentrations of glutathione peroxidase and vitamin E [11, 40, 41].

Glutathione (GSH) is one of the most important antioxidant defenses against oxidative stress and exists in both the reduced (GSH) and oxidized state (GSSG; glutathione disulphide); oxidised glutathione is accumulated inside the cells and the ratio of GSH/GSSG is a good measure of oxidative stress of an organism [9, 39, 42, 43]. The main role of glutathione is as cofactors of several detoxifying enzymes, participates in amino acid transport through the plasma membrane, scavenges hydroxyl radical and singlet oxygen directly, detoxifying hydrogen peroxide and lipid peroxides by the catalytic action of glutathione peroxidase, and is able to regenerate the most important antioxidants, vitamins C and E [9, 44].

Superoxide dismutase (SOD) is an endogenous enzymatic antioxidant that has shown to protect against programmed cell death. Superoxide radicals formed on both sides of mitochondrial inner membranes are efficiently detoxified by Cu, Zn-SOD (SOD1, localized in the intermembrane space), and Mn-SOD (SOD2, localized in the matrix) [43, 45, 46]. In mitochondria and peroxisomes, finding catalase (CAT), catalyzes the dismutation of H_2_O_2_ to water and oxygen [43].

Vitamin C is an antioxidant hydrophilic of low molecular weight and vitamin E is lipophilic; from the diet obtained both vitamin C and vitamin E. A major lipid soluble antioxidant reported is vitamin E (alpha-tocopherol), effective at protecting against membrane LPO, whereas that ascorbate can act as an efficient antioxidant and scavenge a variety of ROS in vitro [12, 28].

### 2.6. Excitotoxicity

Excessive glutamate receptor activation can induce oxidative stress increase, described by the term excitotoxicity, and play a critical role in epileptic brain damage [47]. Glutamate is the principal excitatory neurotransmitter and its interaction with specific membrane receptors is responsible for many neurological functions; these receptors divided into three major types based on their selective agonist: N-methyl-D-aspartate (NMDA), a-amino-3-hydroxy-5methyl-4-isoxalopropionate (AMPA), and kainate. In the brain ionotropic and metabotropic, receptors mediate the action of glutamate via activation of the NMDAr and play a central role in learning and memory [48]. The NMDAr, mediated by Ca^2+^, activates protein kinase A (PKA), mitogen-activated protein kinase (MAPK), and calcium/calmodulin-dependent protein kinase (CAMK) pathways, which converge at the cyclic-AMP-response element-binding protein (CREB) [27]. The phospholipase A2-dependent activity of Ca^2+^ mediated by glutamatergic receptors liberates arachidonic acid (AA), which generates O^2*∙*−^ through its metabolism by lipoxygenases and cyclooxygenases for eicosanoid formation [49]. In various neurodegenerative disorders, excessive activation of glutamate receptors may induce neuronal injury or death predominantly mediated by excessive influx of calcium into neurons through ionic channels triggered by the activation of glutamate ionotropic receptors [47].

### 2.7. DNA Damage

Endogenous DNA damage, which is incidental to normal cellular metabolism, consists of DNA lesions continually generated by spontaneous decay, depurination, depyrimidination, and deamination; free radicals mediated oxidation and strand breaks and other DNA transactions including erroneous base incorporation, base methylation, and alkylation [50]. Base excision repair (BER) is the major mammalian pathway for repair of oxidatively damaged nuclear and mitochondrial DNA (mtDNA) [51, 52]. It is well known that oxidative stress and ROS cause DNA damage, when repair of DNA damage is insufficient, and then damaged DNA accumulates, especially, in the promoter regions of protein-coding genes, and this can lead to transcriptional disruption of active genes, followed by cellular dysfunction and, ultimately, apoptosis [53]. Moreover, the hydroxyl radicals produced near RNA can easily modify RNA because they are highly reactive and cannot diffuse from their sites of formation; therefore, hydroxyl radical-induced modifications constitute the most varied classes of RNA damage [54].

Oxidative damage to DNA leads to the formation of lesions such as 8-hydroxy-2-deoxyguanosine (8-OHdG) is a hydroxyl radical-damaged guanine nucleotide, excised from DNA by endonuclease repair enzymes, and is the most used biomarker of oxidative DNA alteration [55]. One important target of ROS is the mtDNA due to the close proximity to the electron transport chain and the lack of protective histones [56, 57]. The failure of adaptive responses to ongoing oxidative stress in the brain during epileptogenesis, such as mtDNA repair, could lead to an increase in seizure susceptibility. An increase in mitochondrial oxidative stress is able to impair the mtBER, which involves a highly coordinated process catalyzed by the sequential actions of the different DNA repair enzymes. The mRNA levels of some of these proteins have been elevated following KA-induced status epilepticus but decreased during chronic epilepsy [58].

### 2.8. Apoptosis

Apoptosis is a physiological process for removing unwanted cells during development and for maintaining tissue homeostasis. Specific morphological and biochemical changes may be characterized as an apoptotic cells, including cell shrinkage, chromatin condensation, and internucleosomal cleavage of genomic DNA [59, 60]. The extrinsic pathway is a common phenomenon induced either by specific insults mediated through death receptors, whereas, in the intrinsic pathway, death signals act directly or indirectly on the mitochondria, resulting in the release of cytochrome c and formation of the apoptosome complex [61, 62].

A combination of ROS production and mitochondrial Ca^2+^ initiates opening of the MPTP, which allows translocation of proapoptotic molecules from the mitochondria to the cytosol, in order to trigger apoptotic cell death. The activation of MPTP creates an open channel across the mitochondrial inner and outer membranes, which permits the free diffusion of cytochrome c release from mitochondria to cytoplasm where it activates caspase-9, which can then activate caspase-3 [19, 25, 63]. Another family of mitochondrial-associated proteins are the Bcl-2; this family of proteins consists of both proapoptotic (Bad, Bax, and Bim) and antiapoptotic (Bcl-2, Bcl-xl, and Bcl-w) members and it is hypothesized that they exert their effects by interacting with or controlling the inner of MPTP opening [61, 64, 65]. Apoptosis-inducing factor (AIF) is another mitochondrial-associated protein that is normally located in the intermembrane space of mitochondria and upon a proapoptotic signal AIF is released from the mitochondria. AIF subsequently migrates to the nucleus and triggers DNA damage and, on the other hand, also participates in the activation of caspase-9 in the cytoplasm [3, 61]. The alternative apoptotic pathway is the external pathway with death receptors and caspase-8 as main players. Both internal and external apoptotic pathways meet at the level of caspase-3, which activates CAD (caspase activated DNase) or DFF40, thereby inducing specific DNA fragmentation and apoptotic cell death [65, 66]. Experimental evidence has demonstrated that apoptosis is associated with signaling pathways and contributes to seizure-induced neuronal death in brain of animal models of epilepsy (Figure 1) [3, 25, 65].

## 3. Oxidative Stress in Epilepsy

Generalized epilepsy is a chronic disorder characterized by recurrent seizures, which can increase the content of ROS and RNS generation in the brain; several human and experimental studies have shown the relationship between epilepsy and oxidative stress. Despite the fact that it is still not known if oxidative stress is a cause or consequence of this pathology, it has been widely mentioned that an increase in free radical generation can lead prolonged seizures which may result in mitochondrial dysfunction in the hippocampus that precede neuronal cell death and cause subsequent epileptogenesis [67]. Animal models of epilepsy have provided inconsistent results concerning alterations in redox status. While no changes in GSH levels were found to increase at 4 h post-SE in the cortex, suggesting that GSH may play a disproportionate role in the cortex but not in the hippocampus during epileptogenesis [68], several studies provide evidence of a decrease in hippocampal redox status following SE [69, 70]. A time-dependent decrease in the GSH/GSSG ratio accompanied by a moderate increase in GPx activity and a decrease in GR activity in hippocampal homogenates and mitochondria, following KA-induced SE, have been also reported [71]. Extensive neuronal death in the CA3 subfield occurs from 2–7 days following KA treatment after the early onset of reported redox changes, suggesting the altered redox status may contribute to seizure-induced neuronal death [72, 73].

Lipid peroxidation (LPO) is a central feature of oxidative stress and occurs through a radical-mediated abstraction of a bisallylic hydrogen atom from either the polyunsaturated *ω*-3 or *ω*-6 fatty acids; the delocalized radical reacts then with O_2_ through radical coupling leading to the formation of lipid peroxyl radicals (LOO^•^). LOO^•^ generates a number of lipid hydroperoxide products such as malondialdehyde (MDA), 4-hydroperoxy-2-nonenal (HPNE), 4-oxo-2-nonenal (ONE), and 4-hydroxy-2-nonenal (HNE) [19, 55, 56]. The studies of mitochondrial dysfunction or oxidative stress in the human brain are limited due to the low tissue availability. However, lipid peroxidation has been used as peripheral markers in experimental animals, since results demonstrated that KA-induced increased seizure susceptibility associated with mitochondrial oxidative stress in the hippocampus due to increased mitochondrial LPO and loss of glutathione homeostasis [74].

Several clinical studies have found a decrease of antioxidant (GPx, CAT, and Cu–Zn–SOD) levels and activity in blood of patients with progressive myoclonic epilepsies, showing that the activity of Cu–Zn–SOD in patients was lower than in controls. [75]. Likewise, another study showed that the erythrocyte GSH, GSH-Px, plasma total antioxidant status (TAS), and vitamin E concentration were lower than in control of refractory epilepsy group [76]. Similar results have been observed in drug-resistant epileptic patients [77] and elevated levels of MDA as markers of oxidative damage in women with epilepsy and also have been reported [78]. Lipid peroxidation and percentage hemolysis have shown that the antioxidant status was low in the blood of epileptic patients compared to controls, improved after treatment, suggesting that free radicals may be implicated in epilepsy [79].

Diverse reports have shown that prolonged seizure activity (status epilepticus; SE) results in oxidative damage involving calcium overload and induction of apoptosis. Differences in the expression of many caspases (2, 3, 6, 7, and 9) have been detected by immunohistochemistry method in human TLE brain samples; the caspases appear to localize within both the cell soma and dendrites, supporting caspase-mediated cleavage of intracellular structural or synaptic proteins [80, 81]. The Bcl-2 and caspase families analyzed in neocortex samples surgically removed from TLE patients with intractable seizures found significantly higher levels of antiapoptotic Bcl-2 and Bcl-xL compared to autopsy controls. The levels of Bcl-xL positively correlated with patient seizure frequency, suggesting that in human TLE has been modulated both by pro- and anti-apoptotic pathways [82]. Similar studies have shown altered expression of Bcl-2, procaspases (2, 6, 7, 8, and 9), and caspases (3, 7, 8, and 9) family genes on hippocampus of patients with intractable TLE [65]. Likewise, correlative analysis with detection of expression of apoptosis-associated genes including bcl-2, p53, bax, fas, and caspase-3 showed that neuronal apoptosis occurs in mesial temporal sclerosis patients with intractable TLE [83]. Moreover, occasional TUNEL positive cells with apoptotic cells were observed in hippocampus of these TLE patients [65].

## 4. Experimental Models

Animal models of seizures and epilepsy have proven useful as a complementary strategy in advancing our understanding of this disease. The experimental models are divided in two main categories: models of seizures and models of epilepsy. The difference between these two groups is that those models of epilepsy are characterized by multiple spontaneous recurrent seizures (TLE, evoked by pilocarpine or kainic acid), whereas models of seizures are characterized by generalized seizures in response to a single exposure to a potent neurotoxin [16]. An ideal model of epilepsy should have the following characteristics: (1) seizures should be as the spontaneous recurrent seizures, (2) seizures should be similar to seizures in humans, (3) the EEG pattern should be similar to related types of epilepsy, and (4) the frequency of seizures should be sufficient to test acute and chronic effects of drugs [15]. Therefore, the experimental model of epilepsy should be analogous to the human seizure state and it should share very similar neuropathological mechanisms.

### 4.1. Kainic Acid

Kainic acid (KA) is a rigid analog of the putative excitatory neurotransmitter glutamate and potent agonist of the AMPA/kainate class of glutamate receptors. KA model of status epilepticus (SE) is one of the most extensively studied seizure models. Systemic or intracerebral injection of KA, which stimulates a subtype of the ionotropic receptor of the neurotransmitter glutamate, can result in sustained epileptic activity in the hippocampus that lasts for hours, followed by a latent seizure-free period of weeks. Preceding the development of spontaneous recurrent focal seizures that begin between 3 and 4 weeks followed by a selective pattern of brain damage similar neuropathological (cytotoxic brain edema, neuronal degeneration and loss, microgliosis and astrogliosis) to human TLE [3, 15, 84, 85].

KA increase ROS production, mitochondrial dysfunction particularly in hippocampus. Oxidative stress and excessive glutamate receptor activation and the ensuing LPO are extensively associated with seizure activity [3, 72, 75, 86, 87]. The pyramidal neurons of the hippocampus are particularly vulnerable to the neuroexcitatory actions of KA due to the activates ionotropic glutamate receptors, which selectively induces excitotoxic cell death in the CA3 and CA1 hippocampal subfields and within the dentate gyrus showing that the vulnerability of neurons to oxidative stress varies from one brain region to another [72, 85, 88]. On the other hand, CA2 pyramidal neurons and dentate granule cells appear to be resistant to damage induced by KA [85]. Likewise, in the hippocampus, astrocytes in the CA1 region under stress conditions, display selective loss of glutamate transport activity, increased mitochondrial ROS generation, and reduced mitochondrial membrane potential [87, 89].

**Figure 2:**
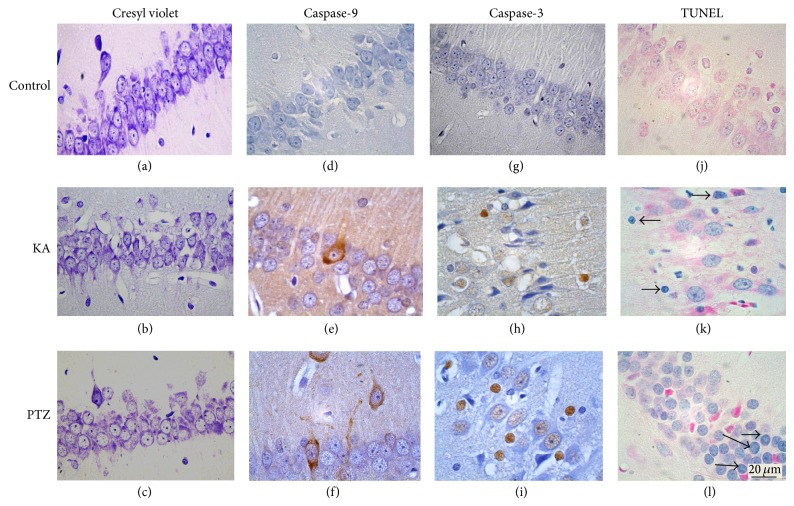
Representative photomicrographs of hippocampal fields of rats at several times after injection of KA or PTZ. Sections stained with cresyl violet, showing neuronal cells in the hippocampus CA1 field (a, b, and c). Hippocampus showing immunoreactive pyramidal cells to caspase-9 (d, e, and f). Immunoreactive cells to caspase-3. The caspase-3 staining was observed in the cytoplasm and nucleus (g, h, and i). Some pyramidal cells (j and k) and granular cells (l) of dentate gyrus were stained positively for TUNEL (↑).

Evidence from animal studies suggests that both brief and prolonged seizures treated by KA can induce activation of caspases and neuronal apoptosis within the hippocampus. DNA fragmentation and chromatin condensation in cerebellar neurons following exposure to KA were reported earlier, demonstrating that KA can induce apoptosis [90]. Similar results (DNA fragmentation) were observed in CA3 pyramidal neurons in hippocampus of rats after focal-onset status epilepticus at 24 h; thus, prolonged seizures can cause apoptosis in hippocampal subfields in addition to the dentate granule cell layer, regardless of model, age, species, and/or strain [91]. Induction of apoptosis also was observed in dentate gyrus neurons of rats with single and intermittent brief seizures induced by KA, suggesting that this process occurring early during epileptogenesis, how primary events in the development of hippocampal pathology [92]. The marked release of cytochrome c from mitochondria into the cytosol and a higher level of caspase-3 cleavage were observed in KA-treated SAM-P8 mice [74]. The cytochrome c release following intrahippocampal KA injection [93], upregulating both caspases 2 and 3 in the rat hippocampus, has been associated with status epilepticusduring the period of epileptogenesis. In our works (data not shown before), we detected by immunohistochemical methods caspase-9 -3 and TUNEL (a marker of irreversible DNA fragmentation) positive cells in hippocampus of rats injected with KA (Figure 2). Related studies have corroborated these results using histochemical (TUNEL or activated caspase-3 staining) or ultrastructural analysis found features of apoptotic cell death present bilaterally in the hippocampus 1–7 days after the elicitation of sustained hippocampal seizure activity by microinjection of KA [65, 93]. Increases of oxidative stress induced by the mitochondrial production of superoxide radicals, increase in LPO, and decreases in GSH resulting from KA administration have been reported; these evidences have shown to play a critical role of oxidative stress on induction of apoptosis neurons in many regions of the brain particularly in the hippocampal regions of CA1 and CA3 [79, 90].

### 4.2. Pentylenetetrazol Model (PTZ)

The Pentylenetetrazol (PTZ) model for induction of epilepsy is considered similar to primary tonic-clonic generalized epilepsy in humans. PTZ is tetrazole derivative with consistent convulsive actions in mice, rats, cats, and primates, when given by the parenteral route and it is considered a GABA selective agonist [94, 95]. It has also been reported that one of the mechanisms that underlie epilepsy produced by PTZ is the increase of voltage at the voltage-gated potassium channel [96, 97]. There is also a known relationship between the imbalance of the inhibitory and excitatory neurotransmission systems, and in the long run, a loss of inhibition mediated by GABA [98]. Specifically, PTZ blocks the GABA_A_ receptor [95, 97] and both GABA_A_ and GABAB_Rs_ are involved in the control of neuronal excitability and epileptogenesis [99]. PTZ initially produces myoclonic jerks, which become sustained, and may lead to waves or polyspikes. On the other hand, the PTZ treatment needs repeated injections to result in cell loss in the hippocampus, which might be a result of enhanced activity of glutamatergic systems [98]. The PTZ treatment leads to hippocampal atrophy in rats shown a selective neuronal loss and astrocytosis [15, 100].

After PTZ induced seizures, significant decreases in GSH, GSSG were reported [101], with reductions in total SOD activity and lipid antioxidant (a-tocopherol) content [102]. MDA, NOS, and lactate dehydrogenase (LDH) had lower levels of SOD [103, 104] and increases of HO^•^ [105] also were observed in several brain regions of PZT-kindled rats. These results suggest that oxidative stress is implicated in PTZ-induced kindling and that antioxidants could play a role in controlling the accompanying changes [103].

On the other hand, Nasser [99] reports for the first time that PTZ-induced seizures triggered activation of caspases-3 to induce widespread apoptotic neuronal death in prenatal rat hippocampal neurons, providing a possible mechanistic link between maternal epilepsy induced neurodegeneration. Likewise, expression caspase-3 and induction of neuronal apoptosis were observed in adult rats induced epileptic seizures with PTZ [106]. We have detected mediated immunohistochemical method of some caspase-9 and caspase-3 positive cells in hippocampus and dentate gyrus in rats exposed to PTZ (Figure 2) and also found occasional TUNEL positive cells in the dentate gyrus in rats treated with PTZ (Figure 2).

### 4.3. Pilocarpine

Systemic administration of the cholinergic muscarinic agonist, pilocarpine, in rats is widely used as an experimental model of status epilepticus because it reproduces many of its features, including refractory seizures, selective interneuron loss, and poor control of seizures by anticonvulsants [107, 108]. Some important features of the pilocarpine model are (i) the induction of acute SE more rapidly than with intraperitoneal (i.p.) KA; (ii) the presence of a latent period followed by the appearance of spontaneous recurrent seizures (SRSs, chronic phase); (iii) the occurrence of widespread lesions some of them localized in the same brain areas affected in TLE patients and associated with neuronal network reorganization in hippocampal and parahippocampal regions; (iv) the fact that seizures are poorly controlled by AEDs in patients and pilocarpine-treated epileptic rodents [95, 109].

The initiation of SE by pilocarpine is due to activation of the cholinergic system, the histopathology, cell loss in the hilus, CA3, and CA1 that leads to a reduction in the Schaffer collateral input, and spontaneous seizure activity is thought to be a result of seizure-induced glutamate release [107]. Experimental evidence has demonstrated that pilocarpine acting through M1 muscarinic receptor subtype, which causes an imbalance between excitatory and inhibitory transmission, results in the generation of SE [110]. Associated with this, an elevation in glutamate levels in the hippocampus maintained the seizures by NMDA receptor activation [111, 112].

Pilocarpine epilepsy model can be mediated by increases in oxidative stress, which could have a role in the hippocampal neurodegeneration. Several reports demonstrated that LPO levels and nitrite content in the brain of adult rats were increased after the acute phase of seizures induced by pilocarpine [113–115]. Agreeing with these results other works have shown in rat hippocampus a significant increase in CAT activity, glutamate content, and a decrease in taurine level [116], as well as the increase of ROS generation in CA1, CA3, and the dentate gyrus [117]. The reduction of the functions of these systems during SE produced by Pilocarpine suggests an involvement of oxidative stress in neuronal death in this experimental epilepsy model.

As discussed above, oxidative stress is a major factor apoptosis induction. The model of epilepsy induced by pilocarpine has shown that it is able to increase the oxidative stress; therefore, the cellular damage observed probably may be due to this mechanism of cell death. However, there is no convincing evidence to show the presence of apoptosis and caspase activation in this model. The necrotic neurons show nuclear pyknosis, chromatin condensation, and internucleosomal DNA fragmentation without nonspecificity of these nuclear changes; these results indicate that, in adult rats, exposed lithium-pilocarpine produces neuronal injury with the appearance of necrosis rather than apoptosis [118]. On the other hand, expression of caspase-3 followed SE showed a significant increase in the number of caspase-3 positive cells in CA1/CA3 area and DG of treated pilocarpine rats [119]. Besides, the activation of caspase-9 and caspase-3 occurred at 4 h, increased into peak levels at 12 h–3 d, and then gradually went down at 7 d–14 d after onset of SE in a mouse pilocarpine model of chronic epilepsy [120]; in another study, the induction of expression of the same caspase-3 appeared at 7 days after lithium-pilocarpine administration [121].

## 5. Conclusions

This review provides an overview of evidence from experimental models that suggests the role of oxidative stress and mitochondrial dysfunction on apoptosis induction in seizure-induced neuronal damage. Oxidative stress enzymes induce a variety of cellular problems that can lead to mitochondrial dysfunction, and accumulation of ROS/RNS not only contributes to the injury of macromolecules such as lipids, proteins, but also affects bioenergetics, glutamate excitotoxicity, and the DNA, with induction of apoptotic signals (Figure 1). A wide variety of models have been developed in order to explore the principal mechanisms of epilepsies. Animal studies have demonstrated that both status epilepticus and recurrent seizures can alter the brain, and the evidence supports that prolonged seizures invoke mitochondrial dysfunction and oxidative stress, leading to caspases activation and induction to apoptosis; therefore, both mitochondrial alteration and oxidative stress are a component of epileptogenesis. Continued research from a complete comprehension of the basic mechanisms of epilepsy, more effective drugs and treatments will be developed for the type of epileptic seizures.
